# Prediction of Extubation readiness in extremely preterm infants by the automated analysis of cardiorespiratory behavior: study protocol

**DOI:** 10.1186/s12887-017-0911-z

**Published:** 2017-07-17

**Authors:** Wissam Shalish, Lara J. Kanbar, Smita Rao, Carlos A. Robles-Rubio, Lajos Kovacs, Sanjay Chawla, Martin Keszler, Doina Precup, Karen Brown, Robert E. Kearney, Guilherme M. Sant’Anna

**Affiliations:** 1 Department of Pediatrics, Division of Neonatology, Montreal Children’s Hospital, McGill University, 1001 Boul. Décarie, room B05.2714. Montreal, Quebec, H4A 3J1 Canada; 2 Department of Biomedical Engineering, McGill University, Montreal, Quebec, H3A 2B4 Canada; 3 Department of Neonatology, Jewish General Hospital, Montreal, Quebec, H3T 1E2 Canada; 4 Division of Neonatal-Perinatal Medicine, Hutzel Women’s Hospital, Wayne State University, Detroit, MI 48201 USA; 5 Department of Pediatrics, Women and Infants Hospital of Rhode Island, Brown University, Providence, RI 02905 USA; 6 Department of Computer Science, McGill University, Montreal, Quebec, H3A 0E9 Canada; 7 Department of Anesthesia, Montreal Children’s Hospital, McGill University Health Center, Montreal, Quebec, H4A 3J1 Canada

**Keywords:** Extubation readiness, Clinical predictors, Cardiorespiratory behavior, Heart rate variability, Respiratory variability, Biomedical signal processing

## Abstract

**Background:**

Extremely preterm infants (≤ 28 weeks gestation) commonly require endotracheal intubation and mechanical ventilation (MV) to maintain adequate oxygenation and gas exchange. Given that MV is independently associated with important adverse outcomes, efforts should be made to limit its duration. However, current methods for determining extubation readiness are inaccurate and a significant number of infants fail extubation and require reintubation, an intervention that may be associated with increased morbidities. A variety of objective measures have been proposed to better define the optimal time for extubation, but none have proven clinically useful. In a pilot study, investigators from this group have shown promising results from sophisticated, automated analyses of cardiorespiratory signals as a predictor of extubation readiness. The aim of this study is to develop an automated predictor of extubation readiness using a combination of clinical tools along with novel and automated measures of cardiorespiratory behavior, to assist clinicians in determining when extremely preterm infants are ready for extubation.

**Methods:**

In this prospective, multicenter observational study, cardiorespiratory signals will be recorded from 250 eligible extremely preterm infants with birth weights ≤1250 g immediately prior to their first planned extubation. Automated signal analysis algorithms will compute a variety of metrics for each infant, and machine learning methods will then be used to find the optimal combination of these metrics together with clinical variables that provide the best overall prediction of extubation readiness. Using these results, investigators will develop an Automated system for Prediction of EXtubation (APEX) readiness that will integrate the software for data acquisition, signal analysis, and outcome prediction into a single application suitable for use by medical personnel in the neonatal intensive care unit. The performance of APEX will later be prospectively validated in 50 additional infants.

**Discussion:**

The results of this research will provide the quantitative evidence needed to assist clinicians in determining when to extubate a preterm infant with the highest probability of success, and could produce significant improvements in extubation outcomes in this population.

**Trial registration:**

Clinicaltrials.gov identifier: NCT01909947. Registered on July 17 2013.

Trial sponsor: Canadian Institutes of Health Research (CIHR).

**Electronic supplementary material:**

The online version of this article (doi:10.1186/s12887-017-0911-z) contains supplementary material, which is available to authorized users.

## Background

### Scope of the problem

Approximately 15,000 infants are admitted to the neonatal intensive care unit (NICU) in Canada each year, of which 11% are extremely preterm (gestational age (GA) ≤ 28 weeks) [1]. Due to lung immaturity, weak respiratory drive and surfactant deficiency, the majority of these infants require endotracheal intubation and invasive mechanical ventilation (MV) during their first days after birth [2]. In a recent large epidemiological study, 85% of extremely preterm infants required MV at some point during hospitalization, most of whom were intubated in the delivery room [3]. Amongst infants with GA of 24 and 25 weeks, 99% and 95% required MV, respectively [3]. Therefore, MV remains an integral part of respiratory management of extremely preterm infants.

Although life-saving at first, prolonged MV has been linked to several adverse outcomes, including ventilator-associated pneumonia, airway trauma and bronchopulmonary dysplasia (BPD) [4]. BPD is the most serious pulmonary morbidity, having been associated with long-term respiratory and neurodevelopmental impairments [5], as well as important social and economic burdens [6]. The duration of MV is a strong predictor for developing BPD; each additional week increases the odds of BPD by a factor of 2.7 [7]. Consequently, clinicians make every attempt to limit its duration and advocate for extubation as early as possible [8]. However, premature extubation carries its own hazards, including lung derecruitment, compromised gas exchange, inspiratory muscle fatigue and ultimately the need for reintubation [9–11]. Indeed, rates of extubation failure in extremely preterm infants have been reported in the literature to be anywhere from 10% to 70%, depending on the population studied and the time frame or criteria used to define failure [12, 13].

Extubation failure increases morbidities and mortality for several reasons [9, 14]. Not only are endotracheal intubations technically challenging [15], but they may be associated with hypoxemia, bradycardia, fluctuations in blood pressures as well as changes in cerebral function [16, 17]. In a recent prospective cohort study, 40% of intubations were associated with adverse events, and 9% of intubations were associated with severe sequelae including hypotension, chest compressions, pneumothorax and death [17]. Furthermore, reintubations risk traumatic injury to the upper airway, lung atelectasis and infection [4, 18, 19]. Together, these complications may lead to cardiorespiratory and/or neurological injuries that may result in long term disability. In fact, emerging studies suggest that reintubation may be an independent risk factor for death or BPD in this population [20, 21]. These observations are very concerning, and underscore the need for lowering the rates of extubation failure while minimizing the duration of MV.

### Predictors of Extubation readiness in preterm infants

Although neonatology has seen major advances in MV and post-extubation respiratory support, the scientific basis for determining whether a patient is ready for extubation remains imprecise. The decision to extubate is usually based on clinical judgment, taking into account personal experience and bedside observation of blood gases, oxygen requirements and ventilator settings [22]. As a result, there are significant practice variations and a paucity of protocols to streamline management for all components of the peri-extubation process, with decisions often being physician-dependent and not evidence-based [22, 23].

Over the years, several attempts have been made to identify objective prediction tools of extubation readiness in preterm infants. In the late 1980s-1990 for instance, it was common practice for infants to undergo a trial of endotracheal continuous positive airway pressure (CPAP) of 2–3 cmH_2_O for periods of 6 to 24 h [24–26]. Infants were extubated if they had no significant apneas, bradycardias or respiratory acidosis during the trial. However, evidence from a meta-analysis refuted this practice, showing that the trial’s prolonged length and low pressures increased the risk of respiratory failure [27]. Subsequently, investigators turned towards shorter assessment periods during which various clinical and physiological variables were evaluated. Unfortunately, many of these prediction tools are of limited applicability today, since they were performed before routine use of antenatal steroids or surfactant therapy. Moreover, the studies were small, single-center and enrolled very heterogeneous populations. For the most part, measures of tidal volume, minute ventilation, breathing pattern, pulmonary mechanics and diaphragmatic function failed to classify infants into their respective extubation class (success or failure) [28–30]. When prediction tools were found to have favorable sensitivities and specificities, they were not prospectively validated [31], or showed no differences in extubation failure rates when compared to clinical judgment alone [32, 33].

More recently, clinicians have shifted towards the use of short-duration spontaneous breathing trials (SBTs) for the assessment of extubation readiness in extremely preterm infants [22]. The SBT is a bedside procedure that consists of observing changes in heart rate, oxygen saturation (SpO2) and/or oxygen requirements during a short trial of endotracheal CPAP. Although the use of a standardized 30-min SBT has been standard of care for assessing extubation readiness in mechanically ventilated adults [34], the evidence for its use in preterm infants is less compelling. In one study, Kamlin et al. performed a 3-min SBT using endotracheal CPAP of 5–6 cmH_2_O in preterm infants with birth weights (BW) < 1250 g who were deemed ‘ready’ for extubation [35]. The SBT showed a sensitivity of 97% and a specificity of 73% at predicting extubation success, thus it was adopted as standard of care in that institution. However, a follow-up prospective audit of this practice found that routine use of SBTs did not improve weaning times or extubation success rates [36]. In the latest prospective observational study, the validity of a 5-min SBT was evaluated in 49 infants with GA < 32 weeks [37]. The SBT had a high sensitivity and positive predictive value, but limited specificity and negative predictive value.

### Cardiorespiratory variability and prediction of Extubation readiness

Variations in heart rate and respiratory rate have long been known to be influenced by the autonomic nervous system (ANS), with cardiovascular integrity depending on the correct balance between sympathetic and parasympathetic tones [38]. Autonomic dysfunction, as characterized by reduced heart rate variability (HRV), has been linked to increased mortality and cardiovascular disease in adult individuals [39]. Respiratory variability (RV), on the other hand, is reduced in conditions of hypoxia, hypercapnia and inspiratory mechanical loading [40–43]. Similarly, evidence from the adult literature has consistently demonstrated reduced HRV and RV in patients who failed weaning from MV [44, 45].

The role of HRV and RV in predicting disease in newborn infants is not understood as well. However, it has become increasingly attractive over the past years, as recent evidence suggests that loss of HRV precedes the clinical presentation of neonatal sepsis [46]. The potential for cardiorespiratory variability measurements to predict extubation readiness has led our group to explore their usefulness in the extremely preterm population. The first evaluation was conducted as part of a retrospective analysis of respiratory data collected by Kamlin et al., whereby RV indices were computed during a 3-min SBT performed prior to extubation [35]. The combination of RV and clinical response to the SBT predicted successful extubation more accurately than either test alone [47]. However, the study used a pneumotachograph to measure respiration, a tool that has several limitations [48]. For those reasons, we conducted a pilot prospective observational study of 56 preterm infants (BW ≤1250 g) in which cardiorespiratory behavior was obtained from electrocardiogram (ECG) and respiratory inductive plethysmography (RIP) signals that captured respiratory movements from the ribcage and abdomen. Data were collected during 2 periods prior to extubation: a 60-min recording on low ventilatory support followed by a 3-min period on endotracheal CPAP. The primary outcome, extubation failure, was defined as the need for reintubation within 72 h from extubation. The study revealed that HRV was significantly lower in infants who failed their first extubation attempt [49]. In addition, both HRV and RV measures had perfect specificity and PPV, but limited sensitivity and NPV. Nevertheless, a major factor limiting the evaluation of RV was the need for manual, breath-by-breath analysis of the respiratory signals. Manual analysis of respiratory signals is expensive, time consuming, operator-dependent and prone to errors. To circumvent this problem, it became more attractive to use an automated, continuous analysis of respiratory behavior. One such example is AUREA, a robust Automated Unsupervised Respiratory Events Analysis system developed by members of our team [50]. AUREA uses uncalibrated RIP signals to compute a number of respiratory-related metrics that are then used to classify the infant’s respiratory patterns on a sample-by-sample basis. The method is fully automated, completely repeatable, standardized, and requires no human intervention. Importantly, it is more efficient than manual scoring (the most common method of analysis) and is not limited by intra- or inter-scorer variability [50].

AUREA was originally designed for older infants recovering from anaesthesia, but was later extended to support analysis of RIP data from preterm infants [51, 52]. Consequently, we used AUREA to reanalyze the original recorded dataset from the pilot study of 56 preterm infants [53]. Exploring the utility of the metrics computed by AUREA revealed that the variability of two metrics (the instantaneous breathing frequency and ribcage movement) were significantly different between infants who succeeded and failed extubation [53]. All in all, those results indicated that cardiorespiratory signals analyzed using AUREA contained information that could be useful to predict successful extubation. However, AUREA computes many different metrics describing cardiorespiratory behavior on a sample by sample basis. Therefore, it is not straightforward to determine which metrics to use, the criteria to select the samples and how to combine them to obtain the best predictor of extubation readiness. Consequently, we applied machine learning methods to explore how to best combine features of HRV and RV to predict extubation readiness. The best results were obtained using a Support Vector Machine (SVM), an advanced machine learning classifier that uses nonlinear decision boundaries [54]. After combining 17 features computed by AUREA, the SVM produced accurate classifications with an optimal true positive rate greater than 85% and a false positive rate of less than 30% [55]. The results of this pilot study were encouraging and suggested that a classifier with such performance had the potential to reduce extubation failures by 80%.

### Rationale

Both prolonged MV and the need for reintubation are associated with short- and long-term complications. Therefore, it is critical to determine the optimal timing for extubation to minimize the duration of MV while maximizing chances of success. There is promising evidence that analysis of cardiorespiratory signals can provide valuable information into the extubation readiness of extremely preterm infants. We therefore hypothesize that extubation readiness of preterm infants can be determined accurately by using machine learning methods to combine clinical variables along with novel, quantitative and automated measures of cardiorespiratory behavior.

### Objectives

This project aims to develop an automated predictor to help physicians determine when extremely preterm infants are ready for extubation, using the combination of clinical tools along with novel and automated measures of cardiorespiratory variability. The research objectives will be accomplished in this following sequence:

1. Generate a library of clinical data and cardiorespiratory signals in preterm infants prior to extubation;
2. Develop a robust model for prediction of extubation readiness, i.e. referred to as APEX (Automated prediction of extubation readiness);
3. Prospectively validate the clinical utility of this prediction model

## Methods

### Study design

This is a prospective, multicenter observational study aiming to develop an automated prediction tool for extubation readiness in extremely preterm infants. The study design conforms to recommendations by the TRIPOD (transparent reporting of a multivariable prediction model for individual prognosis or diagnosis) statement and the study protocol is reported using the Standard Protocol Items: Recommendations for Interventional Trials (SPIRIT). The SPIRIT checklist is available in Additional file 1.

### Study setting

Five tertiary-level NICU’s in North America are involved: the Royal Victoria Hospital, Jewish General Hospital and Montreal Children’s Hospital (Montreal, Quebec, Canada), Detroit Medical Centre (Detroit, Michigan, USA) and Women and Infants Hospital (Providence, Rhode Island, USA). Approval was obtained from each institution’s Ethics Review Board. Enrollment began in September 2013 and is currently ongoing. Of note, the Royal Victoria Hospital and Montreal Children’s Hospital NICU’s merged and moved to a new site in May 2015.

### Eligibility criteria

**Fig. 1:**
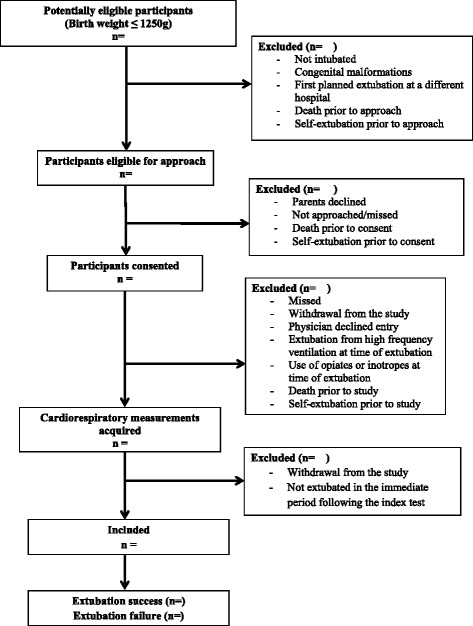
Study enrollment flow diagram template

Figure 1 presents a diagram representing the flow of participants through the study. All infants with BW ≤ 1250 g and requiring MV are eligible for the study. Infants are excluded if they have any major congenital anomalies, congenital heart disease, cardiac arrhythmias, or are receiving any vasopressor or sedative drugs at the time of extubation. Infants are also excluded if they are extubated from high frequency ventilation, or directly to room air, oxyhood or low-flow nasal cannula. Details of all inclusion and exclusion criteria are summarized in Table 1.

**Table 1:** Inclusion and exclusion criteria

Inclusions	Exclusions
Birth weight ≤ 1250 g	Major congenital anomalies
Requiring intubation/mechanical ventilation	Congenital heart disease
First planned extubation	Cardiac arrhythmias
	Receiving any vasopressor at time of extubation
	Receiving any sedatives at time of extubation
	Extubation from high frequency ventilation
	Direct extubation to room air, oxyhood or low flow nasal cannula
	Accidental/unplanned extubation
	Death prior to extubation

### Extubation

There is no consensus on when an extremely preterm infant should be extubated. Thus, prior to initiation of the study, we proposed the following guidelines to consider a patient ‘ready’ for extubation: For infants <1000 g - mean airway pressure (MAP) ≤ 7 cmH_2_O and fraction of inspired oxygen (FiO_2_) ≤ 0.3; For infants ≥1000 g - MAP ≤8 cmH_2_O and FiO_2_ ≤ 0.3. Nevertheless, all decisions regarding weaning, determination of extubation readiness and post-extubation management are ultimately made by the responsible physician. In general, all units have adopted SpO_2_ target ranges according to their respective institutional guidelines and have been practicing a permissive hypercapnia ventilator strategy. Caffeine therapy is commonly administered prior to extubation as part of standard care. Infants typically receive post-extubation respiratory support in the form of either nasal CPAP or non-synchronized nasal intermittent positive pressure ventilation (NIPPV), at the discretion of the attending physician. These are the two most frequently used and best regarded support modalities [22, 23]. However, since design of the study and beginning of patient recruitment, we have observed that an increasing number of infants are being extubated to heated humidified high flow nasal cannula (HHHFNC) therapy. This modality is the subject of ongoing investigations, and some uncertainty remains regarding its effectiveness in preventing extubation failures in the extremely preterm population when compared to CPAP or NIPPV [56]. This has led to adjustment of the final sample size in order to account for this new practice (see ‘sample size calculation’ below).

### Interventions

The development of APEX (the automated prediction model of extubation readiness) involves the following steps: acquisition of cardiorespiratory and clinical data, offline analysis of all the data, derivation and prospective validation of the model. All phases of APEX development are described below.

I. *Acquisition of cardiorespiratory data.*

Infants are studied prior to their first planned extubation, once deemed ‘ready’ by the attending neonatologist. The following cardiorespiratory signals are acquired: (1) ECG using 3 ECG leads placed on the infant’s chest or limbs; (2) Chest and abdominal movements using uncalibrated RIP with the Respitrace QDC system® (Viasys® Healthcare, USA). One RIP band is placed around the infant’s chest at the level of the nipple line, and the other band around the infant’s abdomen, above the umbilicus; (3) SpO_2_ and photoplethysmograph (PPG) signals with a pulse oximeter (Radical, Masimo Corp, Irvine, LA.) placed on the infant’s hand or foot.

**Fig. 2:**
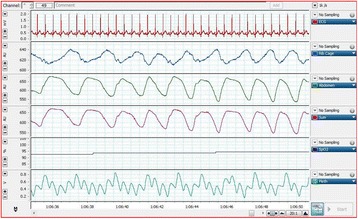
Representative example of a cardiorespiratory recording from a preterm infant. The signals displayed, from top to bottom, are: electrocardiogram, rib cage movements, abdominal movements, sum of rib cage and abdominal movements, oxygen saturation and photoplethysmography

All signals are amplified, anti-alias filtered at 500 Hz, and sampled at 1 kHz by a portable analog-digital data acquisition system (PowerLab version 7.3.8, ADInstruments, Dunedin, New Zealand, © 2009) mounted on a battery-powered laptop computer. Fig. 2 shows a representative example of the signals acquired.

Data is acquired from each infant while quiet, stable and in supine position, during 2 continuous recording periods immediately preceding extubation:

1. A 60-min period while the infant receives any mode of conventional MV. These data will be used to characterize the HRV properties of the infant prior to extubation. However, this data may not be suitable for characterizing RV, since the respiratory pattern is still influenced by the ventilator.
2. A 5-min period will be used to record the respiratory parameters. During this time, the ventilation will be switched to endotracheal CPAP at the same positive end-expiratory pressure level used during the first recording period, so that the cardiorespiratory patterns are controlled by the infant.

II. *Acquisition of clinical data.*

The key clinical variables recorded for each infant are summarized in Table 2, and the following respiratory outcomes are recorded while the infant is hospitalized in the NICU:

**Table 2:** Clinical variables to be collected for infants enrolled in the study

Antenatal and maternal variables	Mother age, parity, complications during pregnancy, maternal medications, intra-uterine growth restriction, mode of delivery, multiple birth, use of antenatal steroids, rupture of membranes, use of antibiotics during labor, histological chorioamnionitis.
Infant characteristics pre-extubation	Gender, birth weight, gestational age, Apgar scores (1, 5 and 10 min), cord blood gases, use of surfactant (age, dose), use of antibiotics and caffeine administration prior to extubation (age and dose).
Infant characteristics at time of extubation	Weight at extubation, age and post-conceptional age at extubation, ventilator mode, peak inflation pressure, positive end-expiratory pressure, mean airway pressure, tidal volume, set inspiratory time, ventilator rate, fraction of inspired oxygen (FiO_2_), oxygen saturation and blood gas
Infant characteristics post-extubation	Type of non-invasive respiratory support, interface used, settings, FiO_2_ and blood gas
Primary extubation outcome	Fulfilling extubation failure criteria within 72 h from extubation
Secondary extubation outcomes	- Fulfilling extubation failure criteria up to 14 days after extubation - Need for reintubation at any time point from extubation until death or discharge (including timing and reasons for reintubation)
Other outcome variables	Total duration (in days) of mechanical ventilation, non-invasive respiratory support and of oxygen supplementation, intraventricular hemorrhage, patent ductus arteriosus, necrotizing enterocolitis, postnatal infection (defined as positive culture from the blood, urine or cerebrospinal fluid), need for postnatal steroids, bronchopulmonary dysplasia at 36 weeks post conceptual age (classified as none, mild, moderate or severe), upper airway complications, diuretics at discharge, retinopathy of prematurity and death occurring anytime in the NICU (including timing and cause).

#### Extubation failure in the first 72 h after extubation

This the primary outcome for the development of APEX. Extubation failure is defined by one or more of the following criteria: (a) FiO_2_ > 0.5 to maintain SpO_2_ > 88% or PaO_2_ > 45 mmHg (for 2 consecutive hours); (b) PaCO_2_ > 55–60 mmHg with a pH < 7.25, in two consecutive blood gases done at least 1 h apart; (c) one episode of apnea requiring positive pressure ventilation with bag and mask; (d) Multiple episodes of apnea (≥ 6 episodes/6 h). This information will be collected prospectively from the nursing flow chart and blood gas records.

#### Extubation failure between 72 h and 14 days after extubation

Following the 72-h period after extubation, infants are monitored for presence of extubation failure criteria (as described above) until 14 days post-extubation.

#### Reintubation

This is a secondary outcome measure and is recorded at any time point from extubation until NICU discharge. The timing and reasons for reintubation are collected in detail since the decision to re-intubate is made by the responsible physician. Therefore, the indications for reintubation may differ from the criteria defining extubation failure.

*III. Data analysis.*

The analysis will be developed in 2 phases. Phase 1 will identify and evaluate cardiorespiratory features (metrics or patterns) that differ in infants who succeed/fail extubation. Phase II will use machine learning methods to determine the optimal combination of these features for the derivation of APEX.

### Phase I: Cardiorespiratory features

All signals will be exported to MATLAB™ (The MathWorks, Inc.) format for the following analyses:

*A)* *Respiratory Signal Analysis.* AUREA will be used to describe respiratory activity in terms of a series of metrics that characterize the amplitude, frequency and phase information of the RIP signals on a sample-by-sample basis [52]. These metrics are computed automatically, provide quantitative measures of the respiratory activity and include:

- *Instantaneous respiratory frequency (f*_*max*_ *):* is the frequency in the respiratory band with the most power between 0.4 and 2.0 Hz. [57] It is estimated by passing the RIP signal through a bank of digital, band-pass filters; the central frequency of the filter with the highest output power at each time defines *f*_*max*_ *.* This yields a sample-by-sample estimate with an accuracy of 0.1 Hz, or half the filter pass-band (0.2 Hz). Note that because we use symmetric, two-sided filters, there is no time delay in estimating *f*_*max*_ *.*
- *RMS metric:* extracts the amplitude information of the respiratory signals, and is defined as the sum of the root mean square (RMS) values for the ribcage (RCG) and abdomen (ABD) RIP signals.
- *Pause metric:* is based on the power of regular breathing in either RCG or ABD. Pauses are defined by a lack of respiratory effort, so the RIP signals are expected to have low relative power in the regular breathing band (0.4–2.0 Hz). The pause metric is defined as the ratio of power in the regular breathing band for a short window to the median regular breathing power for the entire record. This metric is close to 1 during regular breathing and lower during pauses.
- *Movement artifact metric:* defined separately for ABD and RCG, compares the power in the movement artifact band (i.e., 0–0.4 Hz) to that in the regular breathing band. It is calculated using the outputs of a filter bank spanning the frequencies 0–2 Hz; each filter has a 0.2 Hz bandwidth. This metric will be close to +1 during regular breathing and shift towards −1 during movement artifacts.
- *Thoraco-abdominal asynchrony metric:* estimates the phase between RC and AB using selectively filtered RIP signals to improve the signal-to-noise ratio. The filtered signals are then converted to binary signals and an exclusive-OR signal is computed, representing the phase relation between RC and AB at each sample [58]. Averaging the resulting signal over a window length *N*_*A*_ yields an asynchrony metric proportional to the phase shift.

**Fig. 3:**
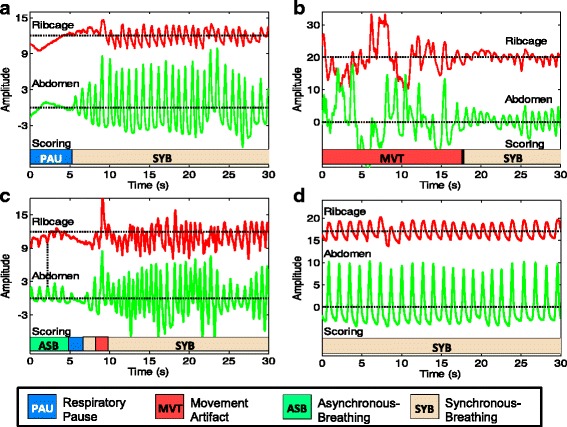
Sample epochs of respiratory data from a preterm infant displaying the respiratory patterns detected automatically by AUREA. AUREA - Automated Unsupervised Respiratory Event Analysis system **a** Pause (PAU), **b** Movement artifact (MVT), **c** Asynchronous breathing (ASB) and **d** Synchronous breathing. Horizontal *dotted* lines indicate the center of each segment

Once the metrics are computed, AUREA then applies k-means clustering to these metrics to assign each time sample of the RIP signals to one of 5 respiratory patterns (also illustrated on Fig. 3):

- Pause (PAU)
- Synchronous-breathing (SYB)
- Asynchronous-breathing (ASB)
- Movement artifact (MVT)
- Unknown (UNK)

The performance of AUREA’s assignment of respiratory patterns will be compared with results of an experienced manual scorer, and fine-tuned accordingly.

*B)* *Heart Rate Analysis.* ECG signals acquired during the recording periods will be analyzed by first converting the ECG signal into a point process by identifying the maxima of the R wave. The resulting signal will then be low-pass filtered using the French-Holden algorithm [59] to generate a continuous HR signal. Instantaneous estimates of power in the: i) Very Low Frequency (VLF) = 0.01–0.04 Hz; (ii) Low Frequency (LF) = 0.04–0.2 Hz, and (iii) High Frequency (HF) = > 0.2 Hz bands will be determined by passing the continuous HR signal through a bank of band-pass filters with appropriate cut-offs. These filters will be implemented in the time domain as symmetric, two-sided finite impulse response filters, making it possible to track changes in HRV as a function of time with no delay.

*C)* *Pulse Oximeter Analysis.* The PPG signal will be analyzed to detect movement artifacts using an algorithm that computes and removes a moving average of the larger quasi-periodic pulse components. The RMS of the residual will be close to zero for clean signals and higher during movement artifacts. This metric is faster and performs better than other methods that use higher order statistics [60, 61]. Oxygen saturation and Pulse Transit Time (PTT) will be computed for artifact-free segments. The PTT estimates the time elapsed between the R-wave of the ECG and the peripheral PPG pulse [62], and has been shown to be useful in the diagnosis of Obstructive Sleep Apnea Syndrome [63].

*D)* *Stationarity.* Each metric is computed for each sample. The behavior of any given sample may vary randomly and/or as a function of time. This will likely occur during the 5-min period on endotracheal CPAP as the infant adapts to a sudden change on respiratory load. Consequently, the time course of each metric will be inspected to ensure that it is stationary. If not, we will first try to break the data set into shorter, quasi-stationary segments. Should this fail, the metric’s time-varying behavior will be described using time series analysis methods.

*E)* *Feature Detection.* We will determine which statistical properties of these metrics describing cardiorespiratory activity are likely to be useful for predicting extubation readiness. To do so, subjects will be separated into two groups, defined by extubation failure or success, and the probability density (PDF) of each metric will be computed and compared. Differences in the variability of a metric will be revealed by changes in the shape of the PDFs; increased variability should result in a broader PDF while a decrease will result in a narrower PDF. In pilot studies, we found that the interquartile range was a useful feature to quantify variability. However, the shapes of the PDFs may suggest other statistics to use as features. The respiratory patterns generated by AUREA, along with the clinical variables collected, will be subjected to a similar analysis. The set of cardiorespiratory and clinical features with discriminative ability will be selected for use with machine learning methods to build the final predictor.

### Phase II: Machine learning

The machine learning phase will examine the hypothesis that subjects ready for extubation can be differentiated from those who are not by using a classifier that combines clinical variables with the features computed in Phase I.

For classification, infants will be assigned to either the SUCCESS or FAILURE groups depending on the primary outcome, extubation failure or success. We will then use discriminative classification algorithms (e.g. SVM [54] and Adaboost [64]) to construct classifiers for risk assessment. SVM is a powerful classification method, which takes existing labeled examples and constructs a non-linear decision boundary providing a class separation. New examples are then classified by comparing them to this boundary. SVM relies on two important insights: the boundary can be defined by the examples that are closest to it (called support vectors) and any new instance can be classified by comparing it to the support vectors. This implicit way of defining the decision boundary permits the use of large numbers of attributes, and the discovery of non-linear relationships between them (rather than simple logical relationships such as “AND” and “OR”). The algorithms to be used provide non-linear classification boundaries as well as a measure of uncertainty in the labeling of each example (expressed as a “margin” between the example and the classification boundary). Unlike other learning algorithms that produce non-linear classifiers, such as neural networks, these algorithms are known to work well with limited numbers of examples, as is the case for our data, and to be very robust to noise in the input features.

*IV. Prospective validation of APEX.*

The development of APEX as described above will use a variety of specialized software tools. These provide the flexibility necessary for exploratory research but may not be suitable for clinical use. Therefore, we will develop an integrated software system that will perform all the data acquisition, signal analysis, and classification operations needed to predict extubation outcome with a user-friendly interface suitable for medical personnel in the NICU. Prototypes of the package will be developed and tested using MATLAB’s interactive environment, which supports all the needed algorithms and provides a complete set of tools for graphical interface development. Once a prototype is available, its clarity and usability will be assessed by recruiting clinicians from the NICUs (neonatologists, respiratory technicians) to test the package in a simulated setting and provide feedback. Once the package is finalized, the MATLAB compiler will be used to generate a stand-alone application that will be installed on the data acquisition machines.

The performance of APEX will then be validated in a prospective study of an additional 50 preterm infants. These will be used only to evaluate the performance of the predictor in the clinical setting. Moreover, the APEX classification algorithm and parameters will be pre-specified and used for all infants. Patient recruitment, acquisition, and follow-up will be the same as for the original study. However, immediately following completion of the cardiorespiratory recordings, APEX will carry out the signal analysis and classification computations to assign the infant to FAILURE, SUCCESS, or UNCERTAIN groups (see ‘Statistical methods’ below). This APEX classification will not be available to the attending staff and so will not influence clinical care.

### Participant timeline

At each NICU, a research coordinator screens all infants for eligibility and maintains a log of all inclusions/exclusions. Parents are approached by a study investigator who is not the attending neonatologist of that baby, and informed parental consent is obtained prior to the first planned extubation. Participants have the cardiorespiratory signals recorded immediately prior to their first planned extubation and clinical information is prospectively collected at various time points from birth until death, discharge or transfer from the NICU, as presented on the SPIRIT participant timeline in Table 3.

**Table 3:**
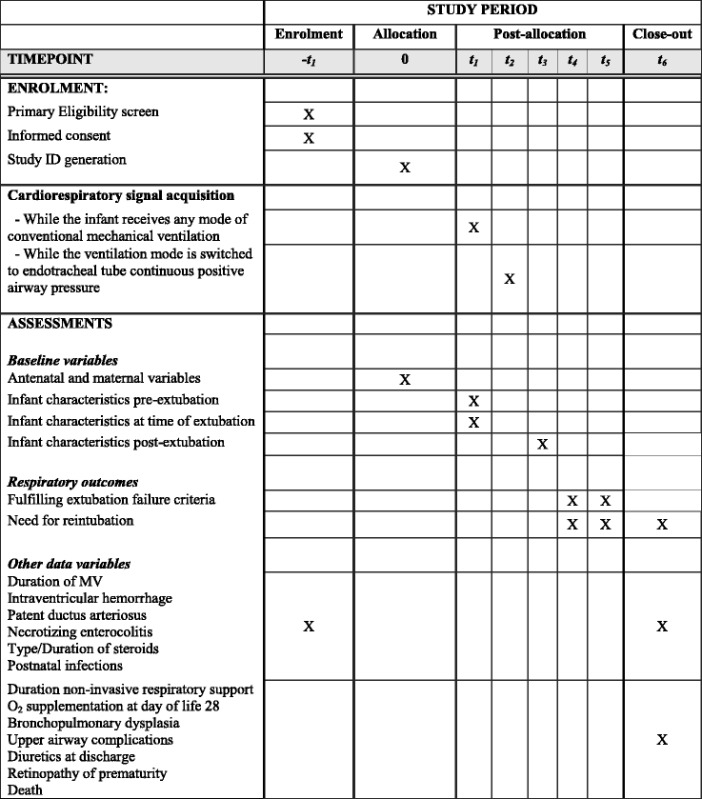
Participant timeline according to the SPIRIT guidelines

-t_1_ = birth to extubation

0 = immediate period prior to initiation of data acquisition

t_1_ = 60-min recording prior to extubation

t_2_ = 5-min recording prior to extubation

t_3_ = immediate period post-extubation

t_4_ = first 72 h period post-extubation

t_5_ = period between 72 h and 14 days post-extubation

t_6_ = discharge, death or transfer from the neonatal intensive care unit

### Sample size

The machine learning methods that will be used for this study have built-in mechanisms to guard against over-fitting the data (i.e., representing the training examples perfectly but having weak predictive power on new data). Consequently, traditional statistical approaches for determining sample size do not apply [65]. Therefore, sample size was estimated by applying a methodology proposed by Obuchowski and McClish and detailed by Zhou et al. [66, 67]. This method relies on estimating the prevalence of the disease of interest in the study population, estimating the variance of the receiver operating characteristics (ROC) curve based on a pilot study, and picking a required precision for the area under the curve (AUC). The prevalence of extubation failure was estimated conservatively to be 20%, based on both a review of the literature and the clinical collaborators’ experience. The variance in the AUC was then estimated by applying bootstrap methods to the data acquired in our pilot study. Using these values and an AUC precision of 0.1 led to an estimated sample size of 170 babies. This sample size would provide a minimum of 5 failure cases in each fold when performing 5-fold cross-validation, thereby ensuring a reliable measurement of generalization power [68]. Nevertheless, in the face of changing practice with the increasing use of HHHFNC post-extubation, and the uncertainty related to its impact on extubation failure rates in this population, the sample size was conservatively increased to 250 patients. As for the prospective validation of APEX, the sample size of 50 infants has been chosen large enough to demonstrate the anticipated benefits and feasibility of the predictor.

### Recruitment

Several strategies have been put in place to ensure steady patient recruitment at each participating site. First, the research coordinators promptly identify eligible patients and approach the parents for consent well before extubation. The coordinators follow the infant’s daily status and proactively organize with the attending physician for the cardiorespiratory recordings to be made prior to extubation. In addition, in order to raise awareness of all NICU personnel (i.e. neonatologists, nurses, respiratory therapists, neonatal nurse practitioners and trainees) about the study, routine activities have been instituted at each unit, in the form of information sessions, in-service training and presentations.

### Data collection methods and data management

In order to harmonize the process of cardiorespiratory acquisition and clinical data collection, assessors from all recruiting sites will get formal training by the same research investigator. Assessors will also receive standardized instructions describing all procedures step-by-step, tips for troubleshooting signal acquisition and definitions of clinical data items. All cardiorespiratory signals will be recorded using a pre-set template from PowerLab’s data acquisition system, thereby ensuring homogeneous sampling methods and a controlled vocabulary of comments added by the investigators during the recording. As for clinical data, it will be entered manually then transcribed into a standardized Microsoft Excel TM (Microsoft Corporation) template that uses multiple layers of quality control to minimize data transcription errors and regulate the type of information entered. Both cardiorespiratory signals and clinical data files will be copied to an encrypted USB key and stored in a locked cabinet that is only accessible to the research investigators. This data will be kept for a period of 7 years after the end of the study, in accordance with the Research Ethics Board guidelines.

Results from the first objective will yield a large, complex dataset that needs to be properly organized, cataloged and readily accessible to investigators from multiple disciplines and geographically-distinct institutions. To facilitate this collaboration, the cloud-based storage and sharing platform Dropbox for Business™ (Dropbox, Inc.) will be used. At the same time, it is important to ensure that the cloud-based file-sharing environment is secure and free of patient identifiers. Thus, our group has developed and implemented an automated anonymization protocol for that purpose, as described in detail elsewhere [69]. In a nutshell, the original cardiorespiratory signals and clinical data are first transferred to a secure repository only accessible to a single administrator responsible for implementing the protocol. The files are then systematically de-identified and automatically transferred to the collaborative repository, where all team members can view the anonymized data in real time [69].

Despite the aforementioned safeguards during the data acquisition process, issues may still arise in the quality of clinical files (e.g. transcription errors, missing data or outlier values) and cardiorespiratory signals (missing or disconnected signals, inadequate recording durations). For those reasons, our group has additionally put in place an algorithm for automated validation and quality control of all files, as described in detail elsewhere [70]. Through automatically-generated summary reports, the completion status of all files is shown and problems are flagged. Moreover, the behavior of various signal properties and clinical variables are described within each site and compared between sites. As a whole, this ensures that all issues are identified and addressed in a timely fashion, that the data quality is uniform across sites and that all included files are validated prior to analysis.

### Statistical methods

#### Machine learning algorithms

The performance of the entire machine learning algorithm (described in ‘data analysis’ above) will be assessed using cross-validation, a standard approach that consists of splitting the data into several sub-sets (‘folds’) while ensuring that the distribution of the data in each subset is similar. Some subsets are used for feature selection and classifier training, while others are used for computing an unbiased estimate of the specificity and sensitivity of the classifiers. Each infant will be assigned to one subset of the data, such that data from the same infant will not be used both for training and testing. We will use stratified 5-fold cross-validation, which ensures that reliable estimates of the sensitivity, specificity, and variance of the predictors can be obtained. ROC curves reflecting the sensitivity and specificity trade-off will be produced and used to analytically determine the best trade-off from the point of view of clinical practice [57].

The machine learning system will produce a binary prediction of whether a baby will succeed or fail extubation. However, for use in the clinical setting, a confidence measure in the classification would be necessary. To this end, we will use a method for estimating conditional probabilities for SVM, proposed by Platt [71], and efficiently implemented by Lin et al. [72]. This approach works on top of an existing support vector machine to produce an estimate of the probability that each example belongs to the class of interest. Our objective is to use these estimates to classify infants into 3 classes: (i) **FAILURE**: infants assigned to the failure group with high confidence; (ii) **SUCCESS**, infants assigned to the success group with high confidence; (iii) **UNCERTAIN**, infants assigned to either the success or fail groups with low confidence. Where the boundary should lie between high and low probability will depend upon the relative cost associated with a false negative (resulting in the extubation of an infant who will fail) versus that of a false positive (extending the period of ventilation for an infant who would otherwise be extubated). Given the nature of the experimental design (i.e. infants are only studied when deemed ready for extubation) we anticipate that the clinical implementation of our methods would involve delaying extubation for infants predicted to fail. We will evaluate performance based on two measures for the FAILURE class, the identification rate (IR) and the false discovery rate (FDR), defined as:

$$ IR=\frac{NF_{fc}}{NF_T};\kern0.39em  FDR=\frac{NS_{fc}}{NS_{fc}+{NF}_{fc}} $$

*where NF*_*T*_ = total number of failures

*NF*_*fc*_ = number of failures assigned to FAILURE class

*NS*_*fc*_ = number of successes assigned to FAILURE class.

Bootstrap methods will be applied to our data to estimate the threshold value that provides the largest value for IR with an acceptable FDR.

#### Prospective APEX validation

The predictive validity of APEX in the clinical context will be evaluated in two ways.

First, we will evaluate the accuracy with which infants are assigned to the high confidence SUCCESS and FAILURE groups by comparing the predicted and observed outcomes. We expect that infants will be assigned to these high-confidence groups with high accuracy. Second, the clinical utility of the approach will also depend on the benefits and costs associated with its potential impact on patient outcome. The **benefits** of using the method can be summarized in terms of the IR, the proportion of extubation failures that could potentially be prevented. The **costs** can be summarized in terms of the FDR, the proportional of infants incorrectly assigned to failure class. Our objectives are to obtain an IR of 0.8 and an FDR of less than 0.5. This would translate into reducing the extubation failure rate from an estimated 20% to less than 5%, at the cost of prolonging the ventilation of one infant for each extubation failure prevented.

### Data monitoring and harms

The leads and bands used to measure cardiorespiratory behavior are non-invasive and come in minimal contact with the baby. Therefore, there are no risks or discomforts associated with the study interventions. Furthermore, none of the study procedures interfere with the standard care that the participating infant will be receiving in the NICU. Any adverse events will be recorded in Case Report Forms and reported to each site’s respective Research Ethics Board in accordance with the protocol and with Good Clinical Practice.

## Discussion

The science of disconnecting extremely preterm infants from the ventilator remains imprecise in today’s NICU. Therefore, such decision continues to be based on subjective evaluations, while clinicians try their best to balance the risks of a failed extubation against the harms of prolonged MV. No accurate predictor of extubation readiness currently exists. For the most part, available predictors are overly simplistic and fail to capture the complex and intrinsic behaviors predisposing infants to a successful extubation. Consequently, the development of an automated tool that could accurately predict successful extubation is extremely important.

To our knowledge, this is the first study to prospectively evaluate clinical and cardiorespiratory behavior of extremely preterm infants prior to extubation. Through multi-disciplinary collaboration between clinicians, biomedical engineers and computer scientists, this project aims to develop a more consistent, comprehensive, and personalized automated tool for the prediction of extubation readiness. The study includes a large sample size, is of multi-center nature and has developed a rigorous framework at all levels of the study design. This will generate the largest database of cardiorespiratory signals and clinical data relating to extubation in extremely preterm infants, therefore providing valuable insight on the complex interactions between all those variables and allowing for investigation of several questions related to this subject.

The study also has some potential limitations. Firstly, all decisions pertaining to weaning from MV, extubation, post-extubation respiratory support and reintubation are made by the responsible physician. This adds significant practice variability and a greater number of confounding factors when developing the prediction model. However, we believe that the pragmatic nature of the study makes it more reflective of clinical reality and therefore more generalizable to the real world. Besides, this concern was addressed in the derivation of the large sample size of patients. Secondly, it is important to note that the prediction model will be developed for infants who were deemed “clinically ready” for extubation by the responsible physician. This results in test-referral bias, whereby only infants pre-selected by the attending physician (based on their own personal bias of extubation readiness) are subjected to the test. Naturally, this leads to a selection of more babies with successful extubation and fewer babies with failed extubation, thereby overestimating sensitivity and underestimating specificity. Therefore, the prediction tool will only be valid in that context and cannot be generalized for all situations, until further validation [73, 74]. Lastly, it is currently unclear which criteria and time frame used to define extubation failure have the most clinical relevance for extremely preterm infants. A recent systematic review of the literature addressed this problem by evaluating extubation failure rates (defined as the need for reintubation) as a function of the time frame used. Amongst infants with BW < 1000 g, cumulative reintubation rates continued to increase up to 7 days post-extubation, with no sign of plateau [13]. Results of this review indicated that a time frame of 72 h could underestimate the true failure rate, and recommended using longer windows of observation in these infants. Although we have defined the primary outcome (extubation failure) as the fulfillment of pre-specified criteria within 72 h from extubation, we are also prospectively evaluating extubation failure using criteria up to 14 days post-extubation, as well as the need for reintubation until discharge.

### Trial status

Enrollment began in September 2013 and is currently ongoing.

## Additional file

Additional file 1: SPIRIT 2013 Checklist: Recommended items to address in a clinical trial protocol and related documents*. (DOCX 60 kb)

## Abbreviations

ABD: Abdomen
ANS: Autonomic nervous system
APEX: Automated prediction of extubation readiness
ASB: Asynchronous-breathing
AUC: Area under the curve
AUREA: Automated unsupervised respiratory event analysis
BPD: Bronchopulmonary dysplasia
BW: Birth weight
CPAP: Continuous positive airway pressure
FiO2: Fraction of inspired oxygen
GA: Gestational age
HHHFNC: Heated humidified high flow nasal cannula
HRV: Heart rate variability
IVH: Intraventricular hemorrhage
MAP: Mean airway pressure
MV: Mechanical ventilation
MVT: Movement artifact
NEC: Necrotizing enterocolitis
NICU: Neonatal intensive care unit
NIPPV: Nasal intermittent positive pressure ventilation
PAU: Pause
PCA: Post-conceptual age
PDA: Patent ductus arteriosus
PDF: Probability density function
PPG: Photoplethysmograph
PTT: Pulse transit time
RCG: Rib cage
RMS: Root mean square
ROC: Receiver operating characteristics
ROP: Retinopathy of prematurity
RV: Respiratory variability
SBT: Spontaneous breathing trial
SpO2: Oxygen saturation
STARD: Standard for reporting of diagnostic accuracy studies
SVM: Support vector machine
SYB: Synchronous-breathing
UNK: Unknown

## References

[1] The Canadian Neonatal Network Annual Report 2015. http://www.canadianneonatalnetwork.org. Accessed April 3^rd^ 2017.

[2] Walsh MC, Morris BH, Wrage LA, Vohr BR, Poole WK, Tyson JE (2005). Extremely low birthweight neonates with protracted ventilation: mortality and 18-month neurodevelopmental outcomes. J Pediatr.

[3] Stoll BJ, Hansen NI, Bell EF, Walsh MC, Carlo WA, Shankaran S (2015). Trends in care practices, morbidity, and mortality of extremely preterm neonates, 1993-2012. JAMA.

[4] Miller JD, Carlo WA (2008). Pulmonary complications of mechanical ventilation in neonates. Clin Perinatol.

[5] Doyle LW, Anderson PJ (2009). Long-term outcomes of bronchopulmonary dysplasia. Semin Fetal Neonatal Med.

[6] McGrath-Morrow SA, Ryan T, Riekert K, Lefton-Greif MA, Eakin M, Collaco JM (2013). The impact of bronchopulmonary dysplasia on caregiver health related quality of life during the first 2 years of life. Pediatr Pulmonol.

[7] Laughon MM, Langer JC, Bose CL, Smith PB, Ambalavanan N, Kennedy KA (2011). Prediction of Bronchopulmonary dysplasia by postnatal age in extremely premature infants. Am J Respir Crit Care Med.

[8] Berger J, Mehta P, Bucholz E, Dziura J, Bhandari V (2014). Impact of early extubation and reintubation on the incidence of bronchopulmonary dysplasia in neonates. Am J Perinatol.

[9] Sant'Anna GM, Keszler M (2012). Weaning infants from mechanical ventilation. Clin Perinatol.

[10] Epstein SK, Ciubotaru RL, Wong JB (1997). Effect of failed extubation on the outcome of mechanical ventilation. Chest.

[11] Rothaar RC, Epstein SK (2003). Extubation failure: magnitude of the problem, impact on outcomes, and prevention. Curr Opin Crit Care.

[12] Hermeto F, Martins BM, Ramos JR, Bhering CA, Sant'Anna GM. Incidence and main risk factors associated with extubation failure in newborns with birth weight < 1,250 grams. J Pediatr (Rio J). 2009;85:397–402. 10.2223/JPED.1922 19690786

[13] Giaccone A, Jensen E, Davis P, Schmidt B (2014). Definitions of extubation success in very premature infants: a systematic review. Arch Dis Child Fetal Neonatal Ed.

[14] Epstein SK, Ciubotaru RL (1998). Independent effects of etiology of failure and time to reintubation on outcome for patients failing extubation. Am J Respir Crit Care Med.

[15] Bismilla Z, Finan E, McNamara PJ, LeBlanc V, Jefferies A, Whyte H (2010). Failure of pediatric and neonatal trainees to meet Canadian neonatal resuscitation program standards for neonatal intubation. J Perinatol.

[16] Shangle CE, Haas RH, Vaida F, Rich WD, Finer NN (2012). Effects of endotracheal intubation and surfactant on a 3-channel neonatal electroencephalogram. J Pediatr.

[17] Hatch LD, Grubb PH, Lea AS, Walsh WF, Markham MH, Whitney GM, et al. Endotracheal Intubation in Neonates: A Prospective Study of Adverse Safety Events in 162 Infants. J Pediatr. 2016;168:62–66.e6. 10.1016/j.jpeds.2015.09.077 PMC4698044 26541424

[18] Torres A, Gatell JM, Aznar E (1995). El-Ebiary M, Puig de la Bellacasa J, González J, et al. re-intubation increases the risk of nosocomial pneumonia in patients needing mechanical ventilation. Am J Respir Crit Care Med.

[19] Venkatesh V, Ponnusamy V, Anandaraj J, Chaudhary R, Malviya M, Clarke P (2011). Endotracheal intubation in a neonatal population remains associated with a high risk of adverse events. Eur J Pediatr.

[20] Manley BJ, Doyle LW, Owen LS, Davis PG (2016). Extubating extremely preterm infants: predictors of success and outcomes following failure. J Pediatr.

[21] Jensen EA, DeMauro SB, Kornhauser M, Aghai ZH, Greenspan JS, Dysart KC (2015). Effects of multiple ventilation courses and duration of mechanical ventilation on respiratory outcomes in extremely low-birth-weight infants. JAMA Pediatr.

[22] Al-Mandari H, Shalish W, Dempsey E, Keszler M, Davis PG, Sant'Anna G (2015). International survey on periextubation practices in extremely preterm infants. Arch Dis Child Fetal Neonatal Ed.

[23] Shalish W, Sant'Anna GM (2015). The use of mechanical ventilation protocols in Canadian neonatal intensive care units. Paediatr Child Health.

[24] Kim EH, Boutwell WC (1987). Successful direct extubation of very low birth weight infants from low intermittent mandatory ventilation rate. Pediatrics.

[25] Kim EH (1989). Successful extubation of newborn infants without preextubation trial of continuous positive airway pressure. J Perinatol.

[26] Tapia JL, Bancalari A, Gonzalez A, Mercado ME (1995). Does continuous positive airway pressure (CPAP) during weaning from intermittent mandatory ventilation in very low birth weight infants have risks or benefits? A controlled trial. Pediatr Pulmonol.

[27] Davis PG, Henderson-Smart DJ. Extubation from low-rate intermittent positive airways pressure versus extubation after a trial of endotracheal continuous positive airways pressure in intubated preterm infants. Cochrane Database Syst Rev. 2001;CD001078. 10.1002/14651858.CD001078 11687097

[28] Smith J, Pieper CH, Maree D, Gie RP (1999). Compliance of the respiratory system as a predictor for successful extubation in very-low-birth-weight infants recovering from respiratory distress syndrome. S Afr Med J.

[29] Veness-Meehan KA, Richter S, Davis JM (1990). Pulmonary function testing prior to extubation in infants with respiratory distress syndrome. Pediatr Pulmonol.

[30] Dimitriou G, Fouzas S, Vervenioti A, Tzifas S, Mantagos S (2011). Prediction of extubation outcome in preterm infants by composite extubation indices. Pediatr Crit Care Med.

[31] Vento G, Tortorolo L, Zecca E, Rosano A, Matassa PG, Papacci P (2004). Spontaneous minute ventilation is a predictor of extubation failure in extremely-low-birth-weight infants. J Matern Fetal Neonatal Med.

[32] Bhat P, Peacock JL, Rafferty GF, Hannam S, Greenough A (2016). Prediction of infant extubation outcomes using the tension-time index. Arch Dis Child Fetal Neonatal Ed.

[33] Gillespie LM, White SD, Sinha SK, Donn SM (2003). Usefulness of the minute ventilation test in predicting successful extubation in newborn infants: a randomized controlled trial. J Perinatol.

[34] MacIntyre NR, Cook DJ, Ely EW, Epstein SK, Fink JB, Heffner JE (2001). Evidence-based guidelines for weaning and discontinuing ventilatory support: a collective task force facilitated by the American College of Chest Physicians; the American Association for Respiratory Care; and the American College of Critical Care Medicine. Chest.

[35] Kamlin CO, Davis PG, Morley CJ (2006). Predicting successful extubation of very low birthweight infants. Arch Dis Child Fetal Neonatal Ed.

[36] Kamlin CO, Davis PG, Argus B, Mills B, Morley CJ (2008). A trial of spontaneous breathing to determine the readiness for extubation in very low birth weight infants: a prospective evaluation. Arch Dis Child Fetal Neonatal Ed.

[37] Chawla S, Natarajan G, Gelmini M, Kazzi SN (2013). Role of spontaneous breathing trial in predicting successful extubation in premature infants. Pediatric Pulmonol.

[38] Draghici AE, Taylor JA (2016). The physiological basis and measurement of heart rate variability in humans. J Physiol Anthropol.

[39] Lahiri MK, Kannankeril PJ, Goldberger JJ (2008). Assessment of autonomic function in cardiovascular disease: physiological basis and prognostic implications. J Am Coll Cardiol.

[40] Brack T, Jubran A, Tobin MJ (1997). Effect of elastic loading on variational activity of breathing. Am J Respir Crit Care Med.

[41] Brack T, Jubran A, Tobin MJ (1998). Effect of resistive loading on variational activity of breathing. Am J Respir Crit Care Med.

[42] Jubran A, Grant BJ, Tobin MJ (1997). Effect of hyperoxic hypercapnia on variational activity of breathing. Am J Respir Crit Care Med.

[43] Jubran A, Tobin MJ (2000). Effect of isocapnic hypoxia on variational activity of breathing. Am J Respir Crit Care Med.

[44] Bien MY, Shui Lin Y, Shih CH, Yang YL, Lin HW, Bai KJ (2011). Comparisons of predictive performance of breathing pattern variability measured during T-piece, automatic tube compensation, and pressure support ventilation for weaning intensive care unit patients from mechanical ventilation. Crit Care Med.

[45] Shen HN, Lin LY, Chen KY, Kuo PH, Yu CJ, Wu HD (2003). Changes of heart rate variability during ventilator weaning. Chest.

[46] Fairchild KD, O'Shea TM (2010). Heart rate characteristics: physiomarkers for detection of late-onset neonatal sepsis. Clin Perinatol.

[47] Kaczmarek J, Kamlin CO, Morley CJ, Davis PG, Sant'anna GM (2013). Variability of respiratory parameters and extubation readiness in ventilated neonates. Arch Dis Child Fetal Neonatal Ed.

[48] Keszler M (2011). Leaks cause problems not only in Washington politics! Has the time come for cuffed endotracheal tubes for newborn ventilation?. Pediatr Crit Care Med.

[49] Kaczmarek J, Chawla S, Marchica C, Dwaihy M, Grundy L, Sant'anna GM (2013). Heart rate variability and Extubation readiness in extremely preterm infants. Neonatology.

[50] Robles-Rubio CA, Brown KA, Kearney RE. Automated unsupervised respiratory event analysis. Conf Proc IEEE Eng Med Biol Soc. 2011:3201–4. 10.1109/IEMBS.2011.6090871 22255020

[51] Robles-Rubio CA, Brown KA, Bertolizio G, Kearney RE. Automated analysis of respiratory behavior for the prediction of apnea in infants following general anesthesia. Conf Proc IEEE Eng Med Biol Soc. 2014:262–5. 10.1109/EMBC.2014.6943579 25569947

[52] Robles-Rubio CA, Bertolizio G, Brown KA, Kearney RE (2015). Scoring tools for the analysis of infant respiratory inductive Plethysmography signals. PLoS One.

[53] Robles-Rubio CA, Kaczmarek J, Chawla S, Kovacs L, Brown KA, Kearney RE (2015). Automated analysis of respiratory behavior in extremely preterm infants and extubation readiness. Pediatr Pulmonol.

[54] Cristianini N, Shawe-Taylor J (2000). An introduction to support vector machines and other kernel-based learning methods.

[55] Precup D, Robles-Rubio CA, Brown KA, Kanbar L, Kaczmarek J, Chawla S, et al. Prediction of extubation readiness in extreme preterm infants based on measures of cardiorespiratory variability. Conf Proc IEEE Eng Med Biol Soc. 2012:5630–3. 10.1109/EMBC.2012.6347271 23367206

[56] Wilkinson D, Andersen C, O'Donnell CP, De Paoli AG, Manley BJ (2016). High flow nasal cannula for respiratory support in preterm infants. Cochrane Database Syst Rev.

[57] Aoude A, Kearney R, Brown K, Galiana H, Robles-Rubio C (2011). Automated off-line respiratory event detection for the study of postoperative apnea in infants. IEEE Trans Biomed Eng.

[58] Motto AL, Galiana HL, Brown KA, Kearney RE (2005). Automated estimation of the phase between thoracic and abdominal movement signals. IEEE Trans Biomed Eng.

[59] French AS, Holden AV (1971). Alias-free sampling of neuronal spike trains. Kybernetika.

[60] Krishnan R, Natarajan B, Warren S (2010). Two-stage approach for detection and reduction of motion artifacts in Photoplethysmographic data. IEEE Trans Biomed Eng.

[61] Selvaraj N, Mendelson Y, Shelley KH, Silverman DG, Chon KH. Statistical approach for the detection of motion/noise artifacts in Photoplethysmogram. Conf Proc IEEE Eng Med Biol Soc. 2011:4972–5. 10.1109/IEMBS.2011.6091232 22255454

[62] Foo JYA, Lim CS (2006). Pulse transit time as an indirect marker for variations in cardiovascular related reactivity. Technol Health Care.

[63] Gil E, Bailon R, Vergara JM, Laguna P (2010). PTT variability for discrimination of sleep apnea related decreases in the amplitude fluctuations of PPG signal in children. IEEE Trans Biomed Eng.

[64] Friedman J, Hastie T, Tibshirani R (1998). Additive logistic regression: a statistical view of boosting. Ann Stat.

[65] Wu B, Abbott T, Fishman D, McMurray W, Mor G, Stone K (2003). Comparison of statistical methods for classification of ovarian cancer using mass spectrometry data. Bioinformatics.

[66] Obuchowski NA, McClish DK (1997). Sample size determination for diagnostic accuracy studies involving binormal ROC curve indices. Stat Med.

[67] X-h Z, Obuchowski NA, McClish DK (2002). Statistical methods in diagnostic medicine.

[68] Kohavi R. A study of cross-validation and bootstrap for accuracy estimation and model selection. In International Joint Conference on Artificial Intellience. 1995:1137–43.

[69] Kanbar LJ, Shalish W, Robles-Rubio CA, Precup D, Brown K, Sant'Anna GM, et al. Organizational principles of cloud storage to support collaborative biomedical research. Conf Proc IEEE Eng Med Biol Soc. 2015:1231–4. 10.1109/EMBC.2015.7318589 26736489

[70] Kanbar LJ, Shalish W, Precup D, Brown K, Sant'Anna GM. Kearney RE. Conf Proc IEEE Eng Med Biol Soc. 2016:2504–7. 10.1109/EMBC.2016.7591239 28268832

[71] Platt J, Smola AJ, Bartlett P, Scholkopf B, Schuurmans D (1999). Probabilistic outputs for support vector machines and comparisons to regularized likelihood methods. Advances in large margin classifiers.

[72] Lin H-T, Lin C-J, Weng RC (2007). A note on Platt's probabilistic outputs for support vector machines. Mach Learn.

[73] Whiting P, Rutjes AW, Reitsma JB, Glas AS, Bossuyt PM, Kleijnen J (2004). Sources of variation and bias in studies of diagnostic accuracy: a systematic review. Ann Intern Med.

[74] Tobin MJ (2011). The new irrationalism in weaning. J Bras Pneumol.

